# Long-term outcome of operated Chiari I patients between 2005 and 2020 in Eastern Finland

**DOI:** 10.1007/s00701-024-05999-y

**Published:** 2024-02-28

**Authors:** Samir Moniruzzaman, Aku Kaipainen, Joona Tervonen, Jukka Huttunen, Henna-Kaisa Jyrkkänen, Terhi J. Huuskonen, Susanna Rantala

**Affiliations:** Neurosurgery KUH NeuroCenter, Kuopio University Hospital, Faculty of Health Sciences, School of Medicine, Institute of Clinical Medicine, University of Eastern Finland, Kuopio, Finland

**Keywords:** Chiari malformation type 1, Posterior fossa decompression, Duraplasty, Outcome

## Abstract

**Purpose:**

The purpose of our study was to examine the long-term outcomes of operated Chiari malformation type 1 (CM1) patients and evaluate whether different duraplasty techniques affected outcome after surgery in Kuopio University Hospital catchment area.

**Methods:**

In this retrospective study, a total of 93 patients were diagnosed with CM1 and underwent posterior fossa decompression surgery with or without duraplasty between 2005 and 2020. All patients’ medical records were examined for baseline characteristics, surgical details, and long-term follow-up data after operation.

**Results:**

The mean age of CM1 patients was 25.9 years (SD 19.2 years), with female preponderance 69/93 (73.4%). The mean clinical follow-up time was 26.5 months (SD 33.5 months). The most common presenting symptoms were headache, symptoms of extremities, and paresthesia. Posterior fossa decompression with duraplasty was performed in 87 (93.5%) patients and bony decompression in 6 (6.5%) patients. After surgery, preoperative symptoms alleviated in 84.9% (79/93) and the postoperative syringomyelia regression rate was 89.2% (33/37) of all patients. The postoperative complication rate was 34.4% (32/93), with aseptic meningitis being the most common, 25.8% (24/93). Revision surgery was required in 14% (13/93) of patients. No significant correlation between postoperative outcome and extent of dural decompression, or type of duraplasty performed was found.

**Conclusion:**

This is the largest reported series of surgically treated CM1 patients in Finland. Posterior fossa decompression is an effective procedure for CM1 symptomology. Duraplasty technique had no significant difference in complication rate or long-term outcomes.

## Introduction

Chiari malformation type 1 (CM1) is generally defined radiologically as cerebellar tonsillar herniation of more than 5 mm below the level of the foramen magnum (FM), but this definition has been criticized for its lack of clinical relevance and alternative definitions such as the functional obstruction of the FM by the inferior cerebellum have been proposed [23]. CM1 is associated with a large variety of symptoms—ranging from asymptomatic to severe. The primary treatment for symptomatic CM1 is the surgical decompression of the posterior fossa, which aims to re-establish cerebrospinal fluid flow and relief pressure on the hindbrain. Indications for surgical treatment of CM1 include persistent symptoms with a confirmed tonsillar herniation and/or the presence of a syrinx [15, 25]. Observation and follow-up are recommended for asymptomatic patients without a syrinx [4].

Surgical strategies for CM1 include posterior fossa decompression without dural opening (PFD) or posterior fossa decompression with duraplasty (PFDD). PFD and PFDD are found to differ regarding the probability of symptom improvement, complication rate, and syrinx resolution [1, 4]. PFDD is slightly favored compared to PFD due to the likelihood of clinical improvement (> 85% versus 80%) and improvement of syringomyelia [17]. PFDD also has a decreased likelihood of recurrence and reoperation rate in the presence of syringomyelia [4, 17, 26]. However, PFDD is associated with a higher rate of overall complications when compared to PFD [26]. PFD is considered an effective approach in pediatric patients as it is thought they have a more distensible dura, allowing for optimal decompression [27]. While PFD is also supported due to its low complication rate, it entails a higher risk of symptom recurrence requiring new surgery [1, 18]. The complication rate has been reported as 2.3–29% in the literature [1, 3, 6, 13, 16, 20, 21, 24, 25]. In a review of 145 operative series, the most common complications include pseudomeningocele, CSF leak, and aseptic meningitis, while surgical mortality was reported as 2–3% [3].

Successful management of CM1 heavily depends on both the correct indication to surgery and surgical technique, which should ultimately be tailored in accordance with the specific anatomical and clinical variations of the individual patient. This includes factors such as bony anatomy of FM, presence of syrinx, and extent of tonsillar herniation [4, 17].

Due to a lack of long-term outcome data regarding CM1 patients in Finland, our aim was to clarify the surgical outcomes of CM1 patients treated in Kuopio University Hospital between 2005 and 2020. Finland has a well-organized healthcare system with accessible, detailed long-term follow-up data, minimizing the likelihood of patients being lost during follow-up. Additionally, we aimed to examine how different PFDD techniques influenced these outcomes. We hypothesized that PFDD fixed by sutures would result in more favorable outcomes and less CSF-related complications compared to PFDD without sutures.

## Materials and methods

### Catchment population of Kuopio University Hospital

Kuopio University Hospital (KUH) is a publicly funded university hospital, under which the department of neurosurgery provides the defined Eastern Finnish catchment population of approximately 850,000 residents with full-time acute and elective neurosurgical services.

### Study population

This retrospective cohort study utilized a consecutive series approach, involving the screening of electronic patient records to include 93 patients from a defined population. Patients included were diagnosed with Chiari malformation type I and underwent primary FM decompression surgery with a diagnosis of CM1 between January 2005 and December 2020.

One patient who underwent parallel surgery with FM decompression in addition to craniocervical fusion was excluded. Data was collected retrospectively and pseudonymized. The follow-up duration for each patient was up until the last CM1-related contact to KUH neurosurgery department.

### Clinical and treatment variables

The following variables were used in the analyses for patients with CM1:

### Baseline characteristics at primary evaluation of CM1 patients

- Patient demographics (sex and age at surgery)
- Presenting symptoms (headache, symptoms of extremities (including ataxia, clumsiness, limb pain, and muscle weakness), paresthesia, balance disorders, visual symptoms (including diplopia, strabismus, nystagmus), nausea, dysphagia, sleep apnea, and urinary incontinence)
- Magnetic resonance imaging (MRI) data (evaluation of extent of tonsillar herniation, presence of syringomyelia, location of syrinx, presence of hydrocephalus, presence of scoliosis both on preoperative and postoperative MRI)

### CM1 surgical details

- Surgeon
- Date of surgery
- Variables regarding CM1 surgery including suboccipital craniectomy, C1 laminectomy, the second cervical vertebra (C2) laminotomy if needed, dural splitting, opening of the arachnoid, tonsillar coagulation, and type of duraplasty
- Our standard surgical technique involves PFDD with C1 laminectomy utilizing a synthetic substitute. This surgical approach is, however, tailored to preoperative imaging, intra-operative findings, and preference of the surgeon

### Follow-up data after treatment of CM1

- Our standard institutional postoperative follow-up routine includes an MRI 3–6 months after surgery, followed by clinical evaluation by the surgeon
- Postoperative follow-up data included improvement of presenting symptoms defined as a reduction of preoperative symptoms after surgery reported by patients, residual symptoms, duration until recurrence of presenting symptoms, decrease in syrinx size, length of hospital stays, additional postoperative hospital visitations, and surgical mortality
- All postoperative complications consisted of the following: bacterial meningitis, aseptic meningitis, pseudomeningocele, hydrocephalus, wound complications (including CSF leak, wound dehiscence, superficial wound infection)
- Revision surgery data included wound revision in operative room, shunt procedures, endoscopic third ventriculostomy (ETV)

### Statistical analysis and artwork

**Fig. 1:**
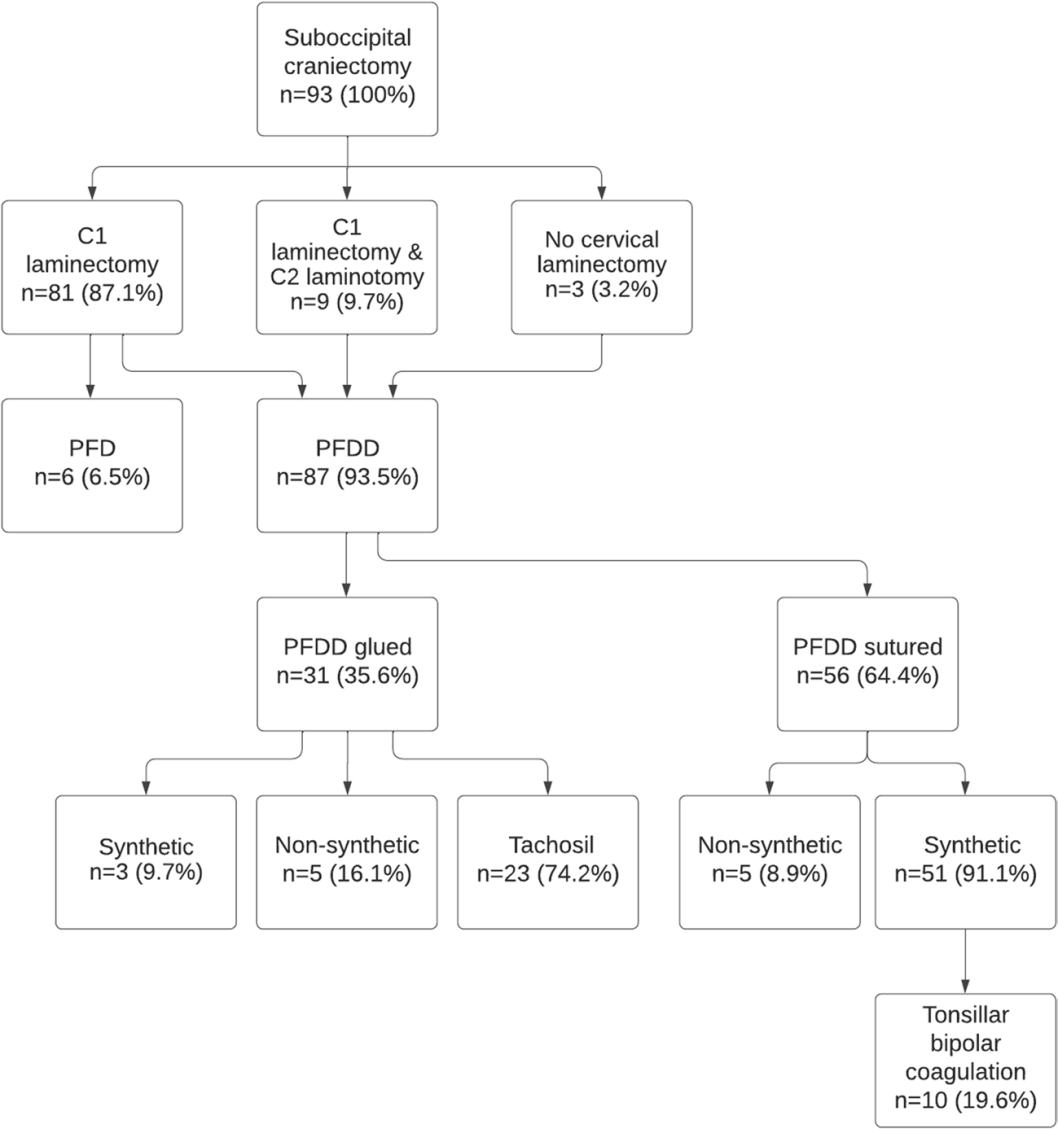
Flowchart illustrating operative details of Chiari I patients. PFD, posterior fossa decompression without duraplasty; PFDD, posterior fossa decompression with duraplasty. Synthetic, Neuro-Patch. non-synthetic, Dura-Guard, and Durepair

**Fig. 2:**
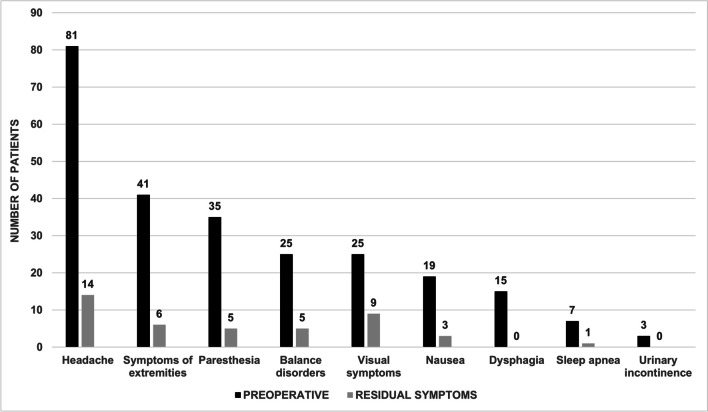
Comparison between preoperative and postoperative occurrence of symptoms

**Fig. 3:**
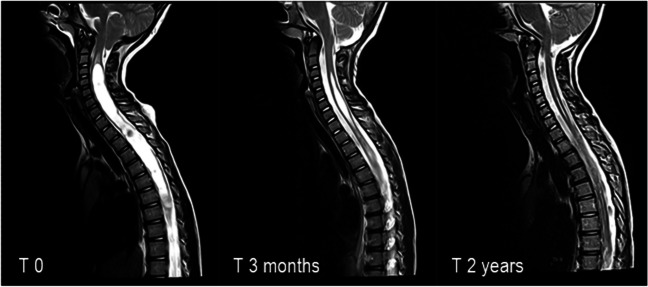
Sagittal view of T2-weighted MR images without contrast, obtained from a pediatric patient with Chiari malformation type I with holocord syrinx from C2 vertebra to conus terminalis. Image on the left illustrates tonsillar herniation and syrinx prior to surgery. Image in the middle was obtained 3 months postoperatively, displaying a decrease in syrinx size. Image on the right displays almost complete syrinx resolution of approximately 2 years postoperatively

All continuous variables were presented as means with standard deviations (SD), and categorical variables were presented using frequencies and percentages. Student’s *t*-test was performed to analyze continuous variables, while Pearson’s *χ*^2^ or Fisher’s exact test was performed to analyze categorical variables. A *P*-value of less than or equal to 0.05 was considered statistically significant. Figure 1 was created with Lucidchart (www.lucidchart.com) and Figs. 2 and 3 with Microsoft Office 365.

### Ethical approval

Approval from the Kuopio University Hospital Ethics Committee was obtained prior to data collection (approval number 5772669).

## Results

### Clinical characteristics of CM1 patients

Between January 2005 and December 2020, 93 patients were surgically treated for CM1 at KUH by nine surgeons. Majority of patients were females (74.2%), with a mean age of 26.1 years (SD 19.2 years) at surgery. A total of 35 (37.6%) patients were under 16 years old. The most common preoperative symptoms at presentation were headache (87.1%), symptoms of the extremities (44.1%), and paresthesia (37.6%). All demographics are reported in Table 2 and preoperative symptoms in Table 1.

### Radiological findings of CM1 patients

The mean tonsillar herniation was 13.9 mm (SD 5.4). The degree of tonsillar herniation in 3 (3.2%) patients was not found due to insufficient radiological data. Syringomyelia was found in 37 (39.8%) of patients, with a syrinx most often (51.4%) located in the cervicothoracic region. Scoliosis was found in 10 (10.8%) patients. Hydrocephalus was treated in two patients (2.2%) before CM1 surgery. One patient had congenital hydrocephalus and underwent ventriculoperitoneal (VP) shunt surgery, while another with communicating hydrocephalus, likely due to FM crowding, underwent endoscopic third ventriculostomy (ETV) procedure. All 93 (100%) patients had preoperative MRI, while 81 (87.1%) patients also underwent a postoperative MRI. Some of the postoperative MR images were missing due to surgeon’s preference to evaluate the patients only clinically. All the MRI findings are summarized in Table 1.

**Table 1:** Postoperative outcomes and complications with a comparison between sutured and non-sutured duraplasty groups

	Overall (*n* = 93)	Duraplasty: sutured (*n* = 56)	Duraplasty: non-sutured (*n* = 31)	*p* value
Improvement of preoperative symptoms	79 (84.9%)	50 (89.3%)	23 (74.2%)	0.077
Headache	67/81 (82.7%)	39/47 (83.0%)	22/28 (78.6%)	0.636
Symptoms of extremities	35/41 (85.4%)	22/24 (88.0%)	11/15 (73.3%)	0.180
Paresthesia	30/35 (85.7%)	19/22 (86.4%)	7/9 (77.8%)	1.00
Balance disorders	20/25 (80.0%)	13/15 (86.7%)	6/9 (66.7%)	0.326
Visual symptoms	16/25 (64%)	7/9 (77.8%)	8/13 (61.5%)	0.648
Nausea	16/19 (84.2%)	11/13 (84.6%)	5/6 (83.3%)	1.00
Dysphagia	15/15 (100%)	7/7 (100%)	5/5 (100%)	NA
Sleep apnea	6/7 (85.7%)	5/5 (100%)	1/2 (50%)	0.286
Urinary incontinence	3/3 (100%)	2/2 (100%)	1/1 (100%)	NA
Radiological findings				
Decrease in syrinx size	33/37 (89.2%)	25/27 (89.3%)	8/10 (80.0%)	0.291
Postoperative complications	32 (34.4%)	20 (35.7%)	12 (38.7%)	0.781
Hydrocephalus	6 (6.5%)	3 (5.4%)	3 (9.7%)	0.662
Bacterial meningitis	3 (3.2%)	3 (5.4%)	0 (0.0%)	0.550
Aseptic meningitis	24 (25.8%)	14 (25%)	10 (32.3%)	0.468
Pseudomeningocele	11 (11.8%)	6 (10.7%)	5 (16.1%)	0.511
Wound complications	10 (10.8%)	7 (12.5%)	3 (9.7%)	1.00
Revision surgery	13 (14.0%)	8 (14.3%)	5 (16.1%)	0.761
Wound revision	7 (7.5%)	5 (10.7%)	2 (6.5%)	1.00
ETV	2 (2.2%)	0 (0.0%)	2 (6.5%)	0.124
Shunt placed after CM1 operation	11 (11.8%)	6 (10.7%)	5 (16.1%)	0.511
Ventriculoperitoneal shunt (VP)	8 (8.6%)	5 (8.9%)	3 (9.7%)	1.00
Ventriculoatrial shunt (VA)	1 (1.1%)	1 (1.8%)	0 (0.0%)	1.00
Pseudomeningocele shunt	2 (2.2%)	0 (0.0%)	2 (6.5%)	1.00
Shunt revision	7 (7.5%)	3 (5.4%)	4 (12.9%)	0.545
The statistical differences were compared with χ²-test and fisher’s exact test for categorical and t-test for continuous variables. Patients who underwent only dural-splitting (PFD group) are included in the overall category				

### CM1 operative details

Decompression of the FM was performed for all CM1 patients, including suboccipital craniectomy with variable extent of bony (C1 laminectomy, C2 laminotomy) and dural decompression (dural splitting, duraplasty) and possible tonsillar coagulation. Only suboccipital craniectomy was performed in three (3.2%) patients, C1 laminectomy was performed in 81/93 (87.1%) patients, and C1 laminectomy and additional C2 laminotomy was performed in nine (9.7%) patients. Opening of the arachnoid layer was performed in 81/87 (93.1%) and additional tonsillar coagulation was performed in 10/93 (10.8%) patients.

The majority of patients 56/87 (64.3%) had a sutured duraplasty, of which a synthetic Neuro-Patch was used in 51/56 (91.1%) patients. Sutured non-synthetic materials included Dura-Guard in three (5.4%) patients and Durepair in two (3.6%) patients. Duraplasty was fixed using only glue in 31/87 (35.6%) patients, of which duraplasty with Tachosil and glue enforcement was performed in 23/31 (74.2%) patients. Duraplasty seams were enforced with tissue sealant in 81/87 (93.1%) patients. Only the dural outer layer was split in six (6.5%) patients, who were aged 16 years and above with no presence of syrinx. One patient underwent a cervical syringostomy prior to FM decompression. Variables regarding operative details are presented in Fig. 1.

### Postoperative outcomes

Outcomes and complications were evaluated according to the last available follow-up data to KUH neurosurgery. The mean duration of postoperative hospital stay was 6.0 days (SD 3.87 days). The mean postoperative clinical evaluation by the surgeon occurred in 2.7 months (SD 1.3 months), while the mean overall follow-up period between surgery and last contact to KUH neurosurgery was 25.5 months (SD 32.0 months). No patients were lost during postoperative follow-up. Reduction in headache was seen in 67/81 (82.7%), symptoms of the extremities in 35/41 (88.4%), paresthesia in 30/35 (85.7%), balance disorders in 20/25 (80.0%), nausea in 16/19 (84.2%), and dysphagia in 15/15 (100%) patients. Postoperative outcomes are presented in Fig. 2 and Table 1.

### Postoperative complications

Postoperative complications occurred in 32/93 (34.4%) patients, slightly more often in adult patients 22/58 (37.9%) as compared with pediatric patients 10/35 (28.6%). The most common complication was aseptic meningitis 24/93 (25.8%), followed by pseudomeningocele 11/93 (11.8%), wound complications 10/93 (10.8%), hydrocephalus in six (6.5%) patients, and bacterial meningitis in three (3.2%) patients. Wound complications consisted of CSF leakage in eight (8.6%) patients, wound dehiscence in one patient, and superficial wound infection in one patient. The pathogen in bacterial meningitis was reported as *Staphylococcus epidermidis* in two patients and *Staphylococcus aureus* in one patient. Postoperative complications were more common in females (41% vs. 17%, *χ*^2^ = 4.51, *p* = 0.034). Variables regarding postoperative complications are presented in Table 1.

### The effect of duraplasty technique on postoperative outcome

Fixing method of the dural substitute (sutured versus glued) had no statistically significant difference on improvement of symptoms, decrease in syrinx size, number of postoperative procedures, or rate of complications. In patients who underwent PFDD, improvement of symptoms occurred in 73/87 (83.9%) patients and complications in 32/87 (36.8%) patients. In patients who underwent PFD, improvement of symptoms occurred in all six patients (100%) and no complications were seen. The outcomes of different duraplasty groups are reported in Table 1.

### Outcome of patients with syringomyelia

A decrease in syrinx size was seen in the majority of syringomyelia patients 33/37 (89.2%), but in three (7.9%) patients no change in syrinx size was observed, and a further increase in syrinx size was observed in one (2.6%) patient. Furthermore, a lower complication rate was seen in patients with syringomyelia when compared with CM1 patients without syringomyelia (22% vs. 43%, *χ*^2^ = 4.45, *p* = 0.035).

### Additional hospital admissions and procedures

A total of 38 (40.9%) patients were readmitted to KUH after surgery. Revision surgery was required in 13/87 (13.8%) patients who had PFDD surgery, while no revision surgery was required in the PFD group. All six (6.5%) postoperative hydrocephalus patients were treated with VP shunts. ETV was performed in two (2.2%) postoperative hydrocephalus patients who further required VP shunt treatment. Postoperative pseudomeningocele was treated using pseudomeningocele shunts in two patients, VP shunts in two patients, ventriculoatrial (VA) shunt in one patient. Shunt complications occurred in seven (63.6%) shunted patients. Aseptic or bacterial meningitis was detected in 9/13 (69.2%) patients requiring revision surgery. There was no additional decompression surgery or surgical mortality. Additional hospital visitations and procedures are presented in Table 2.

**Table 2:** Demographic and preoperative data of Chiari I patients and comparison between sutured and non-sutured duraplasty groups

	Total (*n* = 93)	Duraplasty sutured (*n* = 56)	Duraplasty non-sutured (*n* = 31)	*P* value
Patient characteristics				
Mean age in years (SD)	26.1 (19.2)	21.0 (17.6)	32.3 (19.9)	0.008*
Age groups				0.003*
< 16 years	35 (37.6%)	29 (51.8%)	6 (19.4%)	
≥ 16 years	58 (62.4%)	27 (48.2%)	25 (80.6%)	
Sex				0.437
Male	24 (25.8%)	17 (30.4%)	7 (22.6%)	
Female	69 (74.2%)	39 (69.6%)	24 (77.4%)	
Magnetic resonance imaging				
Presence of hydrocephalus	2 (2.2%)	2 (3.6%)	0 (0.0%)	0.536
Mean extent of tonsillar herniation in millimeters (SD)	13.9 (5.4)	13.9 (4.6)	15.0 (6.7)	0.368
Syringomyelia	37 (39.8%)	27 (48.2%)	10 (32.3%)	0.149
Location of syrinx				
Cervical	13 (14.0%)	7 (12.5%)	6 (19.4%)	0.531
Thoracic	4 (4.3%)	4 (7.1%)	0 (0.0%)	0.292
Cervicothoracic	19 (20.4%)	15 (26.8%)	4 (12.9%)	0.179
Holocord	1 (1.1%)	1 (1.8%)	0 (0.0%)	1.00
Presence of scoliosis	10 (10.8%)	10 (17.9%)	0 (0.0%)	0.012*

The statistical differences were compared with *χ*^2^ test and Fisher’s exact test for categorical variables. Patients who underwent only dural splitting (PFD group) are included in the overall category.

### Residual symptoms

A total of six (6.5%) patients experienced postoperative residual symptoms, divided between three (5.4%) patients who underwent sutured PFDD and three (9.7%) patients with non-sutured PFDD. Residual symptoms consisted of chronic headache in four (4.3%) patients, symptoms of the extremities in one patient, and paresthesia in one patient.

### Recurrence of preoperative symptoms

Symptom recurrence occurred in two (2.2%) patients within one year of postoperative control and in total eight (8.6%) patients within 2 years of postoperative control. Recurring symptoms consisted headache in five (5.4%) patients, followed by paresthesia in three (3.2%) patients, balance disorders in two (2.2%) patients, symptoms of the extremities in two (2.2%) patients, and dysphagia in one patient. No significant correlation was found between the recurrence of preoperative symptoms and other variables.

## Discussion

There is a paucity in the literature on which preoperative and intra-operative prognostic factors are associated with postoperative outcome in CM1 population [4, 15, 17]. In addition, the long-term outcome of operated CM1 patients in Finland has not been comprehensively reported. In this study, we aimed to clarify the long-term outcome of CM1 patients treated at KUH neurosurgery clinic over a 16-year period, and further analyze whether the different PFDD techniques affected overall outcome.

In CM1 treatment, the goal of the surgery is to relief the pressure within the posterior fossa and improve the CSF circulation around obliterated FM. This could be done by either performing bony decompression only (PFD) or further by duraplasty (PFDD) [1, 6, 26]. Previous studies have shown that PFDD might have a higher rate of clinical improvement but on the other hand, a higher rate of complications as compared with PFD only [1, 6, 19].

In our cohort, which is, the largest reported series of surgically treated CM1 patients in Finland [15], most of the patients underwent PFDD and no statistically significant factors between the variable extent of dural decompression, or type of duraplasty performed were identified to affect any postoperative outcomes, i.e., complication rate either. This may be due to the relatively small number of patients in the different subgroups. In this study, the majority (84%) of CM1 patients demonstrated overall improvement of preoperative symptoms, which is in line with previous literature, reporting improvement rates of 72–100% following FM decompression [1–3, 6, 13, 15, 16, 20, 21, 24, 25].

Postoperative complication rates were 36.4% in the PFDD group and 0% in the PFD group; however, the PFD group consisted of only 6 patients, which may influence this result. Furthermore, there were no statistical differences in complication rates between different PFDD technique groups. Considering that the patients who underwent PFDD had more complications, the adjustment of different surgical techniques with the characteristics of the individual patient is essential [4, 17] and the use of PFD for well-selected patients should be kept in mind in the future. Furthermore, all patients in the less aggressive PFD group experienced improvement of symptoms, indicating successful patient selection for this subgroup.

In this study, the most common complication after CM1 surgery was aseptic meningitis in 25.8%, which is in line with previous literature [10]. Aseptic meningitis could be caused by exposure of blood and cellular debris to CSF during the operation [10], and a previous meta-analysis found an increased incidence of aseptic meningitis and CSF leak with PFDD compared to PFD [5]. In this study, most of the patients underwent PFDD procedure with opening of the arachnoid, which is reflected in the rate of aseptic meningitis, whereas in the PFD group there were no cases of aseptic meningitis. A previous study of 112 CM1 patients found that the rate of aseptic meningitis is lower in groups with autologous duraplasty, while the use of xenograft predisposed patients to postoperative aseptic meningitis after which they had a 9.8 times increased risk for requiring permanent CSF diversion [10]. They hypothesized that the use of xenograft or synthetic material as duraplasty may increase the risk for aseptic meningitis through immunologic reactions, as they contain more antigenic material compared to autografts [10]. Combining tissue sealants with a dural substitute may also increase the risk for aseptic meningitis and CSF leak; however, the underlying inflammatory mechanisms remain unclear [22]. In our study, PFDD was performed with xenograft (Dura-Guard & Durepair) in 11% patients and synthetic non-biological material (Neuro-Patch) in 62% patients. Moreover, tissue sealant was used in combination with all PFDD techniques. All these factors may explain our rate of aseptic meningitis. No autologous duraplasty material (pericranium, fascia) was used in our patients, but to reduce the rate of aseptic meningitis this should be considered more carefully in the future.

In this cohort, there was no mortality noted and the rate of major complications was low, as there were six (6.5%) cases of postoperative hydrocephalus requiring further shunt or ETV surgery and three cases of bacterial meningitis. Previous studies report a 5–8.7% incidence of hydrocephalus and 6.5–7.4% requirement for CSF diversion after PFDD [12, 28]. Rates of postoperative meningitis after CM1 surgery have been reported between 1 and 8%, with autografts being associated with the lowest and xenografts with the highest rates [14].

No patient in this study required additional FM decompression surgery. However, revision surgery was required due to pseudomeningocele, CSF leak, and hydrocephalus. Treatment of this patient group is challenging, as these CSF-related complications typically occur simultaneously in the same patient requiring for example shunt placement and wound revision. Postoperative aseptic or bacterial meningitis increases the risk for disturbances of CSF circulation and revision surgeries [10]. Moreover, revision surgeries further predispose patients to develop bacterial or aseptic meningitis, as noted in this study.

The presence of a syrinx due to CM1, even in asymptomatic patients, is often an indication for surgical decompression [8, 15]. In our study, all syringomyelia CM1 patients underwent PFDD, as supported by the current literature [4, 17, 26]. We found an 89.2% decrease of syrinx size after surgery, and the duraplasty technique did not significantly affect the outcome. These findings are comparable to previous studies reporting radiological and clinical improvement of syringomyelia in 65–88% of cases following FM decompression [9, 11, 15, 19]. Surprisingly, we found that the CM1 patients with syringomyelia experienced statistically significantly less postoperative complications (22% vs 43%) as compared with CM1 patients without syringomyelia.

The patient selection for surgery may play a role in this result, as accurately identifying the CM1 patients without syringomyelia whose condition will improve with FM decompression remains difficult due to unambiguity and high degree of personal subjectivity with preoperative symptomology, in addition to lack of other objective imaging parameters [7]. Therefore, the selection of CM1 patients without syringomyelia for decompressive surgery who are not susceptible to improvement while predisposing them to surgical complications and additional procedures may occur. Furthermore, the condition of almost all patients with syringomyelia due to CM1 is expected to stabilize or improve following FM decompression and duraplasty [24]. However, in this study, the improvement rate of preoperative symptoms was 84%, indicating successful patient selection. Considering the relatively high rate of postoperative complications with or without syringomyelia, the correct indication to surgery and a patient-tailored choice of surgical techniques are crucial in treatment.

This is a retrospective cohort study from a single neurosurgical center, entailing all the limitations of such a study design. The relatively few numbers of patients in certain groups made it difficult for our analyses to reach statistical significance. A prospective multi-institutional study with long-term follow-up data, using a validated outcome score should be considered.

## Conclusions

In the present study, we found variability in surgical techniques performed for CM1 patients at KUH. The majority of patients underwent PFDD, including all syringomyelia patients. While no significant effect on outcome or complication rates between PFDD techniques were identified, postoperative improvement and complication rates were comparable to those reported in the literature. This study has analyzed the long-term surgical outcome data of CM1 patients treated in Kuopio University Hospital between 2005 and 2020, adding support to the current notion of tailoring the surgical approach to each individual patient.
